# Severe hypomagnesemia and secondary hypocalcemia associated with vonoprazan in a patient with type 2 diabetes

**DOI:** 10.1210/jcemcr/luag007

**Published:** 2026-02-09

**Authors:** Ryoichiro Aotani, Toshiaki Ohkuma, Taiki Higashi, Ichika Ohmura, Tetsuro Ago

**Affiliations:** Department of Medicine and Clinical Science, Graduate School of Medical Sciences, Kyushu University, Fukuoka 812-8582, Japan; Department of Medicine and Clinical Science, Graduate School of Medical Sciences, Kyushu University, Fukuoka 812-8582, Japan; Department of Medicine and Clinical Science, Graduate School of Medical Sciences, Kyushu University, Fukuoka 812-8582, Japan; Department of Medicine and Clinical Science, Graduate School of Medical Sciences, Kyushu University, Fukuoka 812-8582, Japan; Department of Medicine and Clinical Science, Graduate School of Medical Sciences, Kyushu University, Fukuoka 812-8582, Japan

**Keywords:** diabetes, hypocalcemia, hypomagnesemia, potassium-competitive acid blocker, vonoprazan

## Abstract

Hypomagnesemia is a well-known complication of long-term proton pump inhibitor usage; the stronger acid-suppressive effect of potassium-competitive acid blockers (P-CABs) may increase the risk of hypomagnesemia to a similar or greater extent. However, reports of P-CAB-associated hypomagnesemia are scarce. A 78-year-old Japanese man with a history of type 2 diabetes and myocardial infarction, who was receiving low-dose aspirin and long-term vonoprazan, presented with epigastric discomfort. Laboratory testing revealed significant hypocalcemia, hypomagnesemia, and low intact parathyroid hormone levels, which were consistent with functional hypoparathyroidism. Electrocardiogram (ECG) showed QTc prolongation and frequent premature ventricular contractions. Urinary magnesium excretion was low, suggesting impaired intestinal magnesium absorption rather than renal loss. Vonoprazan was discontinued, and magnesium and calcium supplementation was initiated. Electrolyte abnormalities normalized by day 3, and ECG changes subsequently resolved. No recurrence occurred after switching to an H_2_-receptor antagonist. This case demonstrates severe hypomagnesemia and secondary hypocalcemia associated with long-term use of vonoprazan. P-CABs are often used with low-dose aspirin for ulcer prophylaxis against atherosclerotic diseases in patients with diabetes. Clinicians should be aware of hypomagnesemia as an adverse effect of P-CABs and consider periodic monitoring of serum magnesium levels, particularly in high-risk patients, such as those with type 2 diabetes.

## Introduction

Potassium-competitive acid blockers (P-CABs) are acid-suppressing agents that reversibly bind to the H^+^/K^+^-ATPase (proton pump) in gastric parietal cells, blocking the access of potassium ions to potassium-binding sites [1]. Compared with conventional proton pump inhibitors (PPIs), which have been widely used as first-line therapy for acid-related diseases, P-CABs exert a more rapid, potent, and sustained acid suppression [1]. Vonoprazan, the first-in-class P-CAB, was introduced in Japan in 2015; since then, P-CABs have been developed and launched in Asia, and North, Central, and South America [2].

Patients with diabetes have an increased risk of atherosclerotic cardiovascular disease (ASCVD), and low-dose aspirin is recommended for secondary prevention [3]. To reduce the risk of peptic ulcer bleeding associated with low-dose aspirin, Japanese guidelines recommend the use of PPIs or P-CABs [4]. Consequently, P-CABs are now commonly prescribed to high-risk populations, including patients with diabetes. However, long-term safety data regarding the use of P-CABs are limited [1].

Hypomagnesemia is a potential adverse effect of long-term PPI use. In 2011, the U.S. Food and Drug Administration recommended periodic monitoring of serum magnesium levels during prolonged PPI treatment [5]. The underlying mechanism is thought to involve impaired intestinal magnesium absorption, particularly through transient receptor potential melastatin 6 (TRPM6) and transient receptor potential melastatin 7 (TRPM7) channels, which have a pH-dependent function [6]. Given their stronger and more sustained acid-suppressing effect compared with that of PPIs, P-CABs may pose an equal or higher risk of hypomagnesemia. However, reports of hypomagnesemia associated with P-CABs use remain scarce.

Herein, we report a case of severe hypomagnesemia and secondary hypocalcemia associated with long-term vonoprazan use in a patient with type 2 diabetes and established ASCVD, who was treated with low-dose aspirin for secondary prevention.

## Case presentation

**Figure 1:**
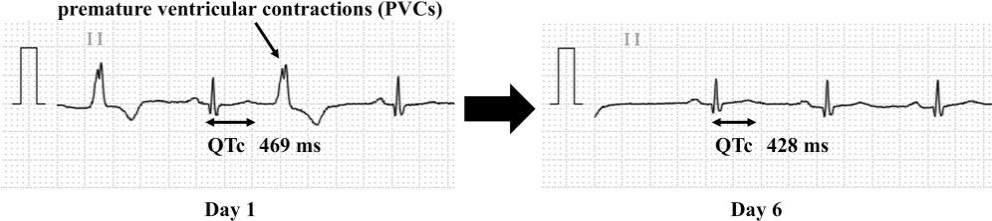
Electrocardiogram findings before and after discontinuation of vonoprazan.

A 78-year-old man with a history of type 2 diabetes and myocardial infarction presented to a local physician with epigastric discomfort. He had been taking low-dose aspirin (100 mg/day) for 9 years for secondary prevention and had been receiving vonoprazan (10 mg/day) for 3 years for ulcer prophylaxis. He had no history of alcohol consumption or smoking. He was referred to our hospital with severe hypocalcemia (6.3 mg/dL [1.6 mmol/L], at the time of referral; normal reference range: 8.8-10.1 mg/dL [2.2-2.5 mmol/L]). On admission, his blood pressure was 131/99 mmHg, heart rate was 68 beats per minute and irregular, body temperature was 36.2 °C, peripheral oxygen saturation was 99% on room air, and consciousness was clear. He was 158.9 cm tall and weighed 56.9 kg (body mass index 22.5 kg/m^2^). No abnormalities were observed during routine physical or neurological examinations. Electrocardiogram revealed a prolonged QTc interval (469 ms) and frequent multifocal premature ventricular contractions (PVCs) (Fig. 1).

## Diagnostic assessment

The initial blood test results (Table 1) showed severe hypocalcemia (albumin-adjusted calcium level, 6.1 mg/dL [1.5 mmol/L]) and hypomagnesemia (0.4 mg/dL [0.16 mmol/L]; normal reference range: 1.8-2.7 mg/dL [0.7-1.1 mmol/L]). An inappropriately low intact parathyroid hormone level was observed, consistent with functional hypoparathyroidism secondary to hypomagnesemia. The estimated glomerular filtration rate was 53 mL/min/1.73 m^2^, excluding severe renal dysfunction. Since there was no evidence of vitamin D deficiency or osteomalacia, and the patient was non-postoperative, showing normal alkaline phosphatase levels and no hypophosphatemia, hungry bone syndrome was considered unlikely. Therefore, we suspected that the hypocalcemia was caused by a relative decrease in PTH level and impaired activity due to hypomagnesemia. Urinary magnesium excretion was low (urinary magnesium, 0.1 mg/dL [0.04 mmol/L]; fractional excretion of magnesium, 0.43%), suggesting impaired intestinal magnesium absorption rather than renal wasting. No diuretics, aminoglycoside antibiotics, platinum-based agents, or calcineurin inhibitors had been administered.

**Table 1:** Laboratory findings on admission

Complete blood count		Reference range	Biochemistry		Reference range
White blood cell	**9180/µL (9.18 × 10^9^/L)**	3300-8600/µL (3.30-8.60 × 10^9^/L)	Total protein	**6.4 g/dL (64 g/L)**	6.6-8.1 g/dL (66-81 g/L)
Red blood cell	**3.45 × 10^6^/µL** **(3.45 × 10^12^/L)**	4.35-5.55 × 10^6/µL (4.35-5.55 × 10^12^/L)	Albumin	**3.6 g/dL (36 g/L)**	4.1-5.1 g/dL (41-51 g/L)
Hemoglobin	**10.8 g/dL (108 g/L)**	13.7-16.8 g/dL (137-168 g/L)	Aspartate aminotransferase	22 U/L (22 U/L)	13-30 U/L (13-30 U/L)
Hematocrit	**32.4% (0.324 L/L)**	40.7-50.1% (0.407-0.501 L/L)	Alanine aminotransferase	14 U/L (14 U/L)	10-42 U/L (10-42 U/L)
MCV	93.9 fL (93.9 fL)	83.6-98.2 fL (83.6-98.2 fL)	Lactate dehydrogenase	**301 U/L (301 U/L)**	124-222 U/L (124-222 U/L)
Platelet	167 × 10^3^/µL (167 × 10^9^/L)	158-348 × 10^3^/µL (158-348 × 10^9^/L)	Alkaline phosphatase	60 U/L (60 U/L)	38-113 U/L (38-113 U/L)
**Endocrinology**			γ-glutamyl transpeptidase	**8 U/L (8 U/L)**	13-64 U/L (13-64 U/L)
Thyroid-stimulating hormone	3.31 µIU/mL (3.31 mIU/L)	0.61-4.23 µIU/mL (0.61-4.23 mIU/L)	Amylase	113 U/L (113 U/L)	44-132 U/L (44-132 U/L)
Free thyroxine	1.67 ng/dL (21.5 pmol/L)	1.00-1.80 ng/dL (12.9-23.2 pmol/L)	Blood urea nitrogen	18 mg/dL (6.4 mmol/L)	8-20 mg/dL (2.9-7.1 mmol/L)
25-Hydroxyvitamin D	**18 ng/mL (45 nmol/L)**	30-100 ng/mL (74.9-249.5 nmol/L)	Creatinine	1.03 mg/dL (91.1 µmol/L)	0.65-1.07 mg/dL (57.5-94.6 µmol/L)
Intact parathyroid hormone	45.1 pg/mL (4.78 pmol/L)	15.0-65.0 pg/mL (1.59-6.90 pmol/L)	Plasma glucose	**122 mg/dL (6.8 mmol/L)**	73-109 mg/dL (4.1-6.1 mmol/L)
**Urinalysis**			Hemoglobin A1c	**6.3% (45 mmol/mol)**	4.9-6.0% (30-42 mmol/mol)
Urine protein	**(1+)**	(−)	C-reactive protein	**0.4 mg/dL (4 mg/L)**	≤0.14 mg/dL (≤1.4 mg/L)
Urine occult blood	(−)	(−)	Sodium	144 mEq/L (144 mmol/L)	138-145 mEq/L (138-145 mmol/L)
Urine creatinine	85.4 mg/dL (7.55 mmol/L)		Potassium	4.5 mEq/L (4.5 mmol/L)	3.6-4.8 mEq/L (3.6-4.8 mmol/L)
Urine sodium	170 mEq/L (170 mmol/L)		Chloride	107 mEq/L (107 mmol/L)	101-108 mEq/L (101-108 mmol/L)
Urine potassium	47.2 mEq/L (47.2 mmol/L)		Calcium	**5.7 mg/dL (1.4 mmol/L)**	8.8-10.1 mg/dL (2.2-2.5 mmol/L)
Urine chloride	173 mEq/L (173 mmol/L)		Inorganic phosphorus	4.4 mg/dL (1.42 mmol/L)	2.7-4.6 mg/dL (0.9-1.5 mmol/L)
Urine calcium	0.2 mg/dL (0.05 mmol/L)		Magnesium	**0.4 mg/dL (0.16 mmol/L)**	1.8-2.7 mg/dL (0.7-1.1 mmol/L)
Urine inorganic phosphorus	41.5 mg/dL (13.4 mmol/L)				
Urine magnesium	0.1 mg/dL (0.04 mmol/L)				
FEMg	0.43%				

Values outside the reference range are shown in bold.

Abbreviations: FEMg, fractional excretion of magnesium; MCH, mean corpuscular hemoglobin; MCHC, mean corpuscular hemoglobin concentration; MCV, mean corpuscular volume.

## Treatment

Based on these findings, we suspected drug-induced hypomagnesemia with secondary hypocalcemia. Vonoprazan was considered the cause of hypomagnesemia and was discontinued on day 1 of admission. Intravenous magnesium sulfate, oral magnesium oxide, intravenous calcium gluconate, and oral calcium lactate, as well as eldecalcitol, an orally active vitamin D_3_ analog (0.5 μg/day, approximately equivalent to standard calcitriol dosing), were administered immediately.

## Outcome and follow-up

**Figure 2:**
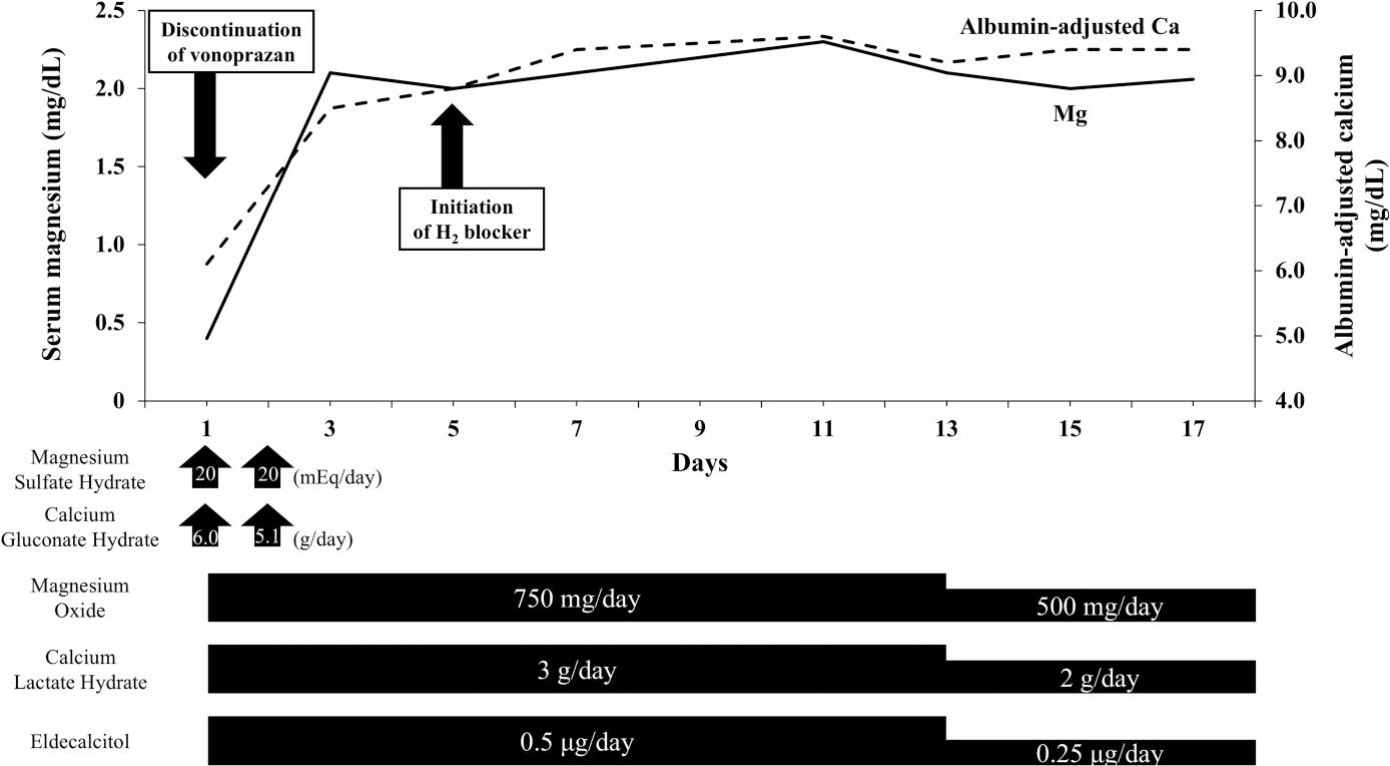
Clinical course of serum magnesium and calcium levels during hospitalization. Reference ranges: serum magnesium, 1.8-2.7 mg/dL (0.7-1.1 mmol/L); albumin-adjusted calcium, 8.8-10.1 mg/dL (2.2-2.5 mmol/L).

As an alternative to acid-suppressive therapy, an H_2_-receptor antagonist was initiated on day 5. By day 3, the serum calcium and magnesium levels had normalized (Fig. 2). The patient's epigastric discomfort and PVCs observed on continuous cardiac monitoring also resolved. A follow-up 12-lead ECG performed on day 6 showed normalization of the QTc interval and resolution of arrhythmia (Fig. 1). Electrolyte supplements were tapered starting on day 13, and the patient was discharged on day 19 without recurrence of electrolyte abnormalities. Because he had pre-existing osteoporosis, an oral active vitamin D_3_ analog was continued after discharge as part of his long-term osteoporosis management, independent of the management of hypomagnesemia. Oral magnesium and calcium supplementation was discontinued within 1 month after discharge.

## Discussion

We report a case of a patient with type 2 diabetes and a history of ASCVD, who experienced severe hypomagnesemia and secondary hypocalcemia after long-term use of vonoprazan while receiving low-dose aspirin for secondary prevention. These abnormalities improved rapidly after vonoprazan treatment was discontinued, and the electrolytes were replaced. Notably, hypomagnesemia did not recur after switching to an H_2_-receptor antagonist, supporting the causal role of vonoprazan. This case underscores the importance of recognizing the potential for clinically important hypomagnesemia as an adverse effect of long-term P-CAB therapy, particularly in high-risk patients such as those with type 2 diabetes and cardiovascular comorbidities requiring ulcer prophylaxis.

Although hypomagnesemia is a recognized side effect of long-term PPI use, it has not been extensively reported for P-CABs use. PPIs are thought to cause hypomagnesemia by impairing intestinal magnesium absorption through inhibition of pH-dependent TRPM6 and TRPM7 channels [6]. P-CABs, which exhibit stronger and longer-lasting acid suppression than PPIs, may also increase the risk of magnesium deficiency. However, reports of hypomagnesemia associated with P-CABs are limited. In a patient with lung cancer receiving carboplatin, severe hypomagnesemia (0.4 mg/dL [0.16 mmol/L]) with torsades de pointes occurred within ∼2 weeks of switching from lansoprazole to vonoprazan [7]. Moreover, severe hypomagnesemia (0.2 mg/dL) and hypocalcemia (albumin-adjusted calcium, 5.7 mg/dL [1.42 mmol/L]) due to >2 years of vonoprazan use was reported in a patient with a history of cardiovascular disease, who was taking clopidogrel and presented with altered consciousness [8]. Furthermore, convulsive seizures due to severe hypomagnesemia (0.4 mg/dL [0.16 mmol/L]) occurred 3 weeks after starting vonoprazan in a patient with ileal diffuse large B-cell lymphoma who underwent ileocecal resection and had a history of PPI use [9]. In contrast, in the present case, the patient developed severe hypomagnesemia and secondary hypocalcemia, accompanied by QTc prolongation and frequent PVCs. The fractional excretion of magnesium was low, indicating impaired intestinal absorption rather than renal wasting, and no other apparent cause of magnesium loss was identified. The rapid improvement in electrolyte abnormalities and ECG changes after discontinuation of vonoprazan further supports a causal relationship. In addition, serum calcium levels normalized after magnesium and calcium supplementation, and no recurrence of hypocalcemia was noted. Given the absence of other apparent causes, hypocalcemia was likely secondary to hypomagnesemia. Magnesium deficiency impairs the secretion and action of parathyroid hormone, contributing to secondary hypocalcemia. Notably, type 2 diabetes is associated with an increased risk of hypomagnesemia, with a prevalence of 10-30% compared with 3-10% in the general population [10], possibly due to insulin resistance. Insulin activates TRPM6 in the distal convoluted tubule; therefore, insulin resistance reduces TRPM6-mediated renal magnesium reabsorption and increases magnesuria [10]. Given this background, the additional inhibitory effect of vonoprazan on intestinal magnesium absorption may further increase the risk of hypomagnesemia in patients with diabetes.

Magnesium is an essential mineral involved in numerous physiological processes, including neuromuscular excitability, cardiac conduction, and glucose metabolism. Hypomagnesemia can cause nonspecific symptoms such as tremors, tetany, and muscle weakness, and may lead to more severe manifestations, including coma, convulsions, and arrhythmias. Furthermore, it is associated with increased risk of mortality [11] and cardiovascular disease [12]. Despite its physiological importance, serum magnesium levels are not routinely measured in clinical settings [10]. The elevated risk of hypomagnesemia in patients with type 2 diabetes, may be exacerbated with long-term use of P-CABs via impairing intestinal magnesium absorption. With the increasing use of P-CABs worldwide, reports of P-CAB-associated hypomagnesemia may become more common in clinical practice. This case adds to the limited literature on vonoprazan-associated hypomagnesemia and underscores the need for regular serum magnesium monitoring, particularly in high-risk populations such as patients with type 2 diabetes receiving long-term P-CAB therapy.

In conclusion, this case highlights the potential for severe hypomagnesemia and secondary hypocalcemia associated with the long-term use of vonoprazan. As type 2 diabetes is a risk factor for hypomagnesemia, the use of potent acid-suppressive agents, such as P-CABs, may further enhance this risk. Clinicians should be aware of this often under-recognized adverse effect and consider periodic monitoring of serum magnesium levels, particularly in patients with type 2 diabetes receiving P-CAB therapy for ulcer prophylaxis.

## Learning points

- Potassium-competitive acid blockers (P-CABs), like proton pump inhibitors, can cause severe hypomagnesemia accompanied by functional hypoparathyroidism and secondary hypocalcemia, leading to electrocardiographic abnormalities.
- Type 2 diabetes increases baseline risk of hypomagnesemia; potent acid suppression with P-CABs may further elevate this risk, particularly in patients with diabetes receiving long-term therapy.
- Periodic monitoring of serum magnesium and calcium levels should be considered in high-risk patients, such as those with type 2 diabetes and established ASCVD who are receiving low-dose aspirin for secondary prevention and long-term vonoprazan therapy for ulcer prophylaxis.

## Data Availability

Data sharing is not applicable to this article as no datasets were generated or analyzed during the current study.
